# An Unusual Tale of Two Appendices in a Case of Acute Appendicitis: One Gangrenous, One Inflamed

**DOI:** 10.7759/cureus.27263

**Published:** 2022-07-25

**Authors:** Virendra K Rathore, Kamakshi M Raveendran, Lakshminarayanan Sadhasivan, Karthikeyan Lakshminarayanan, Rajiv Verma

**Affiliations:** 1 Surgery, Neyveli Lignite Corporation General Hospital, Cuddalore, IND; 2 General Surgery, Government Medical College Patiala, Patiala, IND; 3 General Surgery, Santosh Hospitals, Ghaziabad, IND

**Keywords:** acute appendicitis, diagnosis of acute appendicitis, ruptured appendix, two appendices, appendicular perforation, anamolies of appendix, cave-wallbridge classication, double appendices, duplication of appendix, gangrenous appendicitis

## Abstract

Appendicectomy is one of the most common surgeries performed worldwide. The incidence of acute appendicitis is higher among adolescents and young adults. Though various positions of the appendix, such as retrocecal, pelvic, subcecal, pre- or post-ileal, and their clinical implications have been well established, appendiceal anomalies like duplication or triplication of the appendix are yet to receive attention due to their very low incidence. We report an incidental finding of a duplicated appendix in a 19-year-old girl who presented with features of acute appendicitis. What makes this case report an interesting learning point for young surgeons is the identification of a perforated appendix with gangrene at the tip, along with an inflamed duplicated appendix. This report alerts us to the need for a thorough intraoperative inspection, to look for possible anatomical abnormalities, and to take the right management decisions to avoid unnecessary re-explorations. While operating a patient with features of acute appendicitis, a failure to identify a duplicated appendix is comparable to abandoning an inflamed appendix in-situ. Such instances not only increase the morbidity due to complications like the formation of pelvic abscesses, wound dehiscence, and surgical site infection, but also cause mortality.

## Introduction

The surgical importance of the vermiform appendix lies in its inflammatory propensity known as acute appendicitis and its malignancy at times. Its functional roles are mainly immunological and in maintaining the gut microbiota. The appendix is short and broad at birth. It becomes progressively longer and more tubular during adulthood due to differential growth of the caecum over the years [1]. The appendicular base lies at the convergence of three taeniae at the caecum while the tip is variable (retrocecal 60%, pelvic 30%, retroperitoneal 7% to 10%). Agenesis of the appendix, its duplication, and triplication are rare anomalous variations of the appendix that are reported [2]. The incidence of duplication of the appendix appears to be 0.004% to 0.009%, with less than a hundred cases reported worldwide [3]. Surgeons need to be aware of such anomalous appendices that may account for the differences in clinical presentation as well as operative intervention.

## Case presentation

A 19-year-old girl presented to the Emergency Department of Neyveli Lignite Corporation (NLC) India Limited, General Hospital with complaints of colicky lower abdominal pain for three days and vomiting for one day. There was no history of fever, burning micturition, or menstrual complaints.

On examination, she had a significant tachycardia with a pulse rate of 120 beats/min and the blood pressure was 100/70 mm hg. She was afebrile without pallor, icterus, pedal edema, and lympadenopathy. The abdominal was warm with tenderness and rebound tenderness at McBurney’s point on palpation. She also had considerable guarding and rigidity in the right lower abdomen on deep palpation. The auscultation confirmed normal bowel sounds.

**Figure 1:**
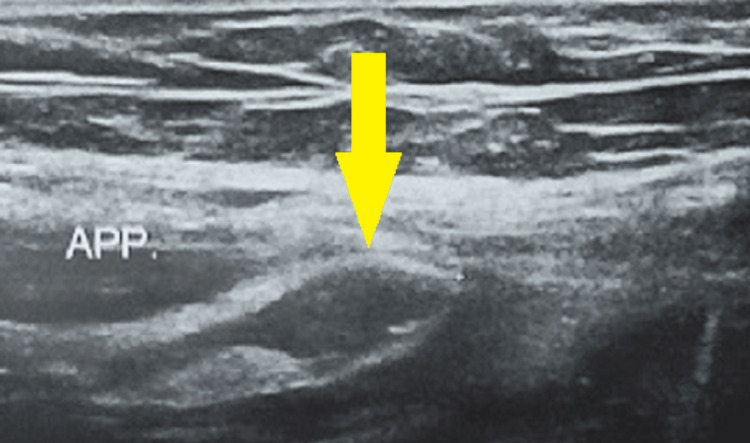
Ultrasonography depicting an inflamed appendix with a 35 mm transverse diameter (arrow) APP.: Appendix

Her blood investigations revealed a leukocytosis of 16,300/cu.mm and polymorphs were 73% in the peripheral smear. Her renal and liver function tests were normal. A urine examination showed enormous pus cells. The chest and abdomen X-rays were normal. Ultrasonography (USG) of the abdomen and pelvis revealed an aperistaltic, tubular, non-compressible, and dilated appendix with a 35 mm transverse diameter with fluid collection in the surrounding area (Figure 1).

**Figure 2:**
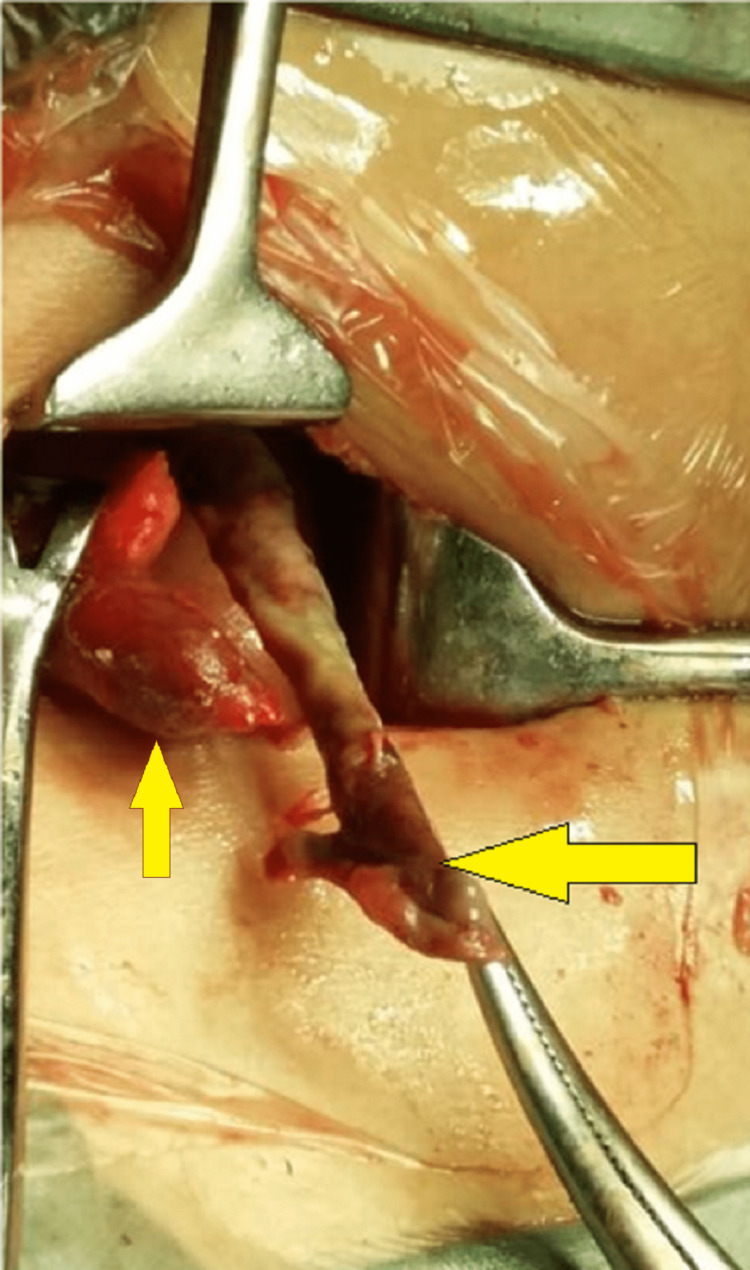
Intraoperative picture showing the two appendices—the inferior appendix in retrocecal position with gangrenous changes and perforation at the tip (side-ward arrow), and the superior appendix on the anterior taenia with inflammation (upward arrow).

**Figure 3:**
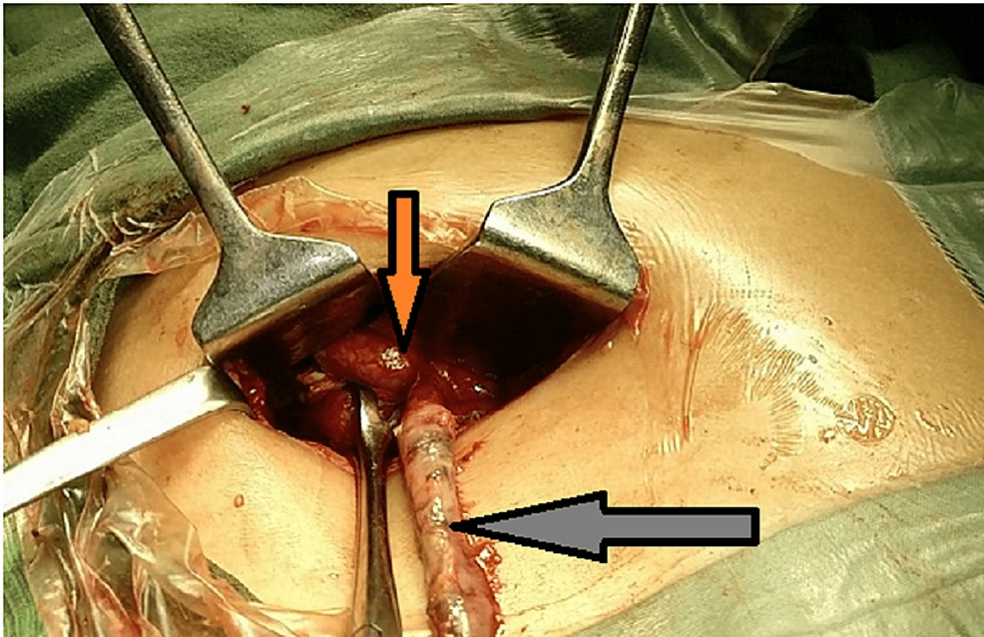
Intraoperative image showing two appendices arising from two different sites (arrows)

**Figure 4:**
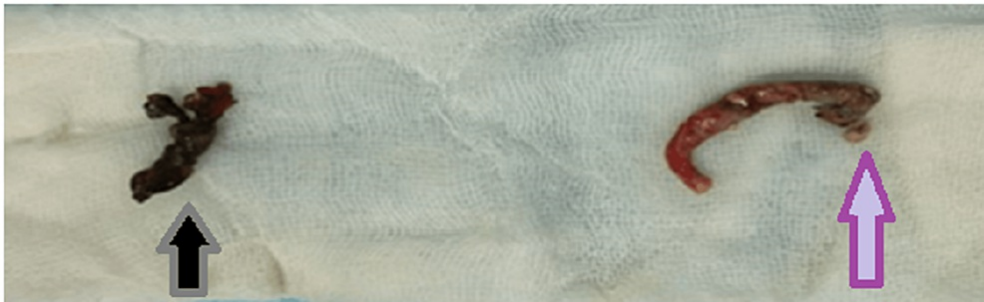
The gross specimens of the two appendices and the fecolith (arrows)

**Figure 5:**
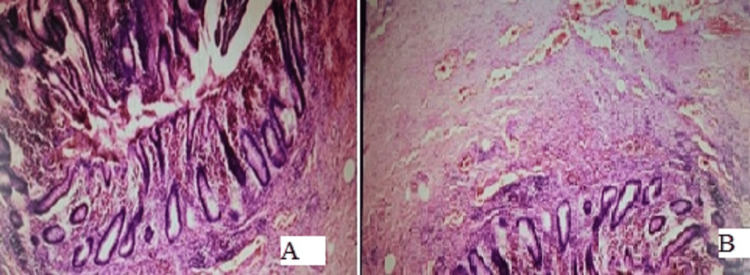
The microscopic histopathological pictures of the two appendices A: The histopathological picture of the inferior appendix found in the retrocecal position depicts the gangrenous changes with transmural inflammation, B: The histopathological picture of the superior duplicated appendix from the anterior taenia reveals inflammatory changes

She was resuscitated with intravenous (IV) crystalloids and was planned for emergency appendicectomy. Preoperative antibiotic prophylaxis was provided. On exploring through a gridiron incision along the McBurney's point, a moderate amount of pus mixed with free fluid was observed. Two appendices were found, one in retrocaecal position at the confluence of taeniae, the other above it, emerging from a single taenia at the anterior aspect of the caecum (Figure 2). The retrocaecal appendix at the confluence of taeniae was gangrenous and perforated with a visible fecolith, while the superior duplicated appendix was inflamed (Figure 3). Both the appendices were transfixed and ligated after control of the appendicular artery. The specimen was sent for histopathological examination (Figure 4). A drain was placed in the wound. The wound was closed in layers after complete hemostasis. The postoperative period was uneventful. The patient recovered well with IV antibiotics and analgesics. Drain removal was done on postoperative day (POD) four and she was discharged on POD five. The histopathological examination revealed features of transmural inflammation in both the appendices (Figure 5).

## Discussion

Appendiceal anomalies are one of the rarest of their kind, with double appendixes having an incidence of 0.004% to 0.009% that are commonly discovered incidentally [4]. The first case of a double appendix was reported by Picoli in 1982 [5]. Though contrast-enhanced computed Ttomography (CT) may rarely be able to help in diagnosing a duplicated appendix, it is difficult for abdominal sonography to identify duplication [6]. Due to the non-availability of CT at the time and with limited resources in our hospital, the clinical diagnosis of acute appendicitis was confirmed with a USG. Triplication of the appendix is an even rarer anomaly with only two cases reported till now [7].

The duplicated appendix was first classified by Cave in 1936 and was modified by Wallbridge in 1962 [6]. The classification is as follows [6]: Type A is described as an incomplete appendicular duplication. Type B is described as one normal appendix from the cecum and another separate appendix, which is subdivided into B1, B2, and B3. The B1 subtype is described as a bird-like anomaly with appendices on either side of the ileocecal valve. The B2 subtype is an appendicular duplication with a second appendix arising from one of the taeniae. While the duplicated appendix arises from hepatic flexure in the case of the B3 subtype, in the B4 subtype it arises from splenic flexure. Type C is described as a duplicated caecum, and type D was included by Mesko in 1986 [6], which is described as a horseshoe appendix with a double opening of the appendix into the caecum. Type B2 is the most common of all these, with no other associated anomalies. Other types are associated with hindgut duplications, colonic atresia, bladder abnormalities, etc. [6]. Previously, in 1941, Waugh classified duplication of the appendix simply as the double barrel type, bird-like type, and taenia coli type [8].

In this case report, type B2 of the duplicated appendix was found as per the modified Cave-Wallbridge classification [6]. A similar case was reported by Anbuchelvam et al., where the inferior appendix was perforated and a duplicated inflamed appendix was found superiorly arising from one anterior taenia [6]. Though appendicular perforation with gangrene was our finding, a duplicated appendix is commonly an incidental finding, as seen in the case reported by Varshney et al. [9]. Acute appendicitis in both the appendices was reported by Baimakhanov et al. and colleagues [10]. Topal reported an incidental appendicular duplication on biopsy [11]. A bifid inflamed appendix of type A Cave-Wallbridge classification was reported by Nabil et al. [12].

Due to its very low prevalence, it is always difficult to identify a duplicated appendix intraoperatively and thereby neglected until specifically looked for. Such was the case described by Travis et al., where duplicated appendicitis was discovered after a previous appendicectomy [13]. Similarly, Sarfraz et al. encountered a patient who developed post-appendicectomy peritonitis due to rupture of the second appendix on POD eight and was subsequently re-operated [14].

An inflamed appendix may serve as a lead point for ileocolic intussusceptions in infants. Aberra reported a case of a six-month-old infant who presented with ileocolic intussusception in which the duplex appendix was the lead point [15]. A case of complete duplication of the appendix with situs inversus totalis was reported by Ngulube et al. in a one-day-old neonate who presented with meconium ileus and imperforate anus [16].

## Conclusions

Appendiceal duplication or triplication contributes to a wide range of morbidity and, in some unfortunate cases, even to mortality following generalized peritonitis and septicemia. When a duplicated appendix is missed inadvertently during an appendicectomy, it may burst out during the early recovery phase and require re-exploration. As adolescents and young adults are the most common victims of acute appendicitis, the inability to identify and manage a duplicated appendix becomes a tragic story, not only for the patient but also for the surgeon. Although the prevalence of duplicated appendix is very low, missed diagnosis may lead to medico-legal issues and waste of resources in terms of re-do surgery, increased morbidity, and even mortality. Improper clamping or trans-fixation of a duplicated appendix may even lead to a catastrophic event like the formation of a colonic fistula. In the case of a duplicated appendix arising from hepatic flexure or splenic flexure, there may be a need for extending the incision intraoperatively, which in turn may add to the morbidity. Excessive bleeding, tissue damage, increased intraoperative time, anesthesia-related complications due to prolonged surgery, postoperative superficial or deep organ space surgical site infection, excessive postoperative pain, prolonged hospitalization, re-exploration, enterocutaneous fistula, etc. are the most dreaded scenarios speculated in the event of neglecting a duplicated appendix.

Though imaging modalities may help in identifying appendiceal anomalies to an extent, a vigilant intraoperative examination is key to avoiding complications associated with mismanagement. A diligent clinical examination and a meticulous intraoperative assessment to rule out anomalies are the ways to solve such cases in areas with no access to modern technology, particularly in the developing and underdeveloped world. Therefore, it is essential for young surgeons to have a thorough knowledge of rare gastrointestinal abnormalities. The management of a duplicated appendix consists of proper identification and ligation of both appendiceal bases, followed by an inspection to rule out associated anomalies. The literature about such rare cases always plays an important role in the learning process of surgeons as well as radiologists. 
